# ACCEPTABILITY OF THE CONFIDENCE INTERVENTION: INITIAL RESULTS FROM SATISFACTION SURVEYS AND QUALITATIVE INTERVIEWS

**DOI:** 10.1093/geroni/igad104.2002

**Published:** 2023-12-21

**Authors:** Susanna Mage, Donna Benton, Kylie Meyer

**Affiliations:** University of Southern California, Los Angeles, California, United States; University of Southern California, Los Angeles, California, United States; Case Western Reserve University, Cleveland, Ohio, United States

## Abstract

Evaluation of an intervention’s acceptability with end-users during the pilot stage can identify barriers to future implementation. Determination of acceptability is particularly important for the CONFIDENCE intervention, as there are no rigorously tested financial wellbeing interventions among family caregivers to persons living with dementia. As a part of the CONFIDENCE pilot study, investigators are using survey techniques and qualitative interviews with participants to assess intervention acceptability. Thus far, N=9 caregivers have completed satisfaction surveys and N=3 participated in a Zoom-based, one-on-one interview. Descriptive statistics were applied to evaluate preliminary satisfaction survey data. Interview transcripts underwent preliminary thematic analysis by the first author. The codebook was guided by the Theoretical Framework of Acceptability (TFA), which posits seven domains of acceptability (e.g., self-efficacy). Results suggest overall acceptability of the intervention. All caregivers who completed the satisfaction survey “Agreed” (3; 33%) or “Strongly agreed” (6; 67%) that they would recommend the CONFIDENCE program to other caregivers. Within specific domains, most caregivers found content easy to understand (8; 89%), easy to access (7; 88%), and convenient to attend (7;78%). The thematic analyses reinforced these findings of acceptability. (“I came into the program right when I needed it.”) Four aspects of the TFA emerged: 1) clear understanding of the intervention and how it works, 2) perception that the intervention was effective in achieving its purpose, 3) confidence in the ability to perform the program requirements, and 4) overall positive affective attitude about the intervention experience. “Every piece of information was helpful,” shared one caregiver.

